# Giant Idiopathic Pulmonary Artery Aneurysm: An Interesting Incidental Finding

**DOI:** 10.1155/2014/251373

**Published:** 2014-03-09

**Authors:** Afsoon Fazlinejad, Mohammad Vojdanparast, Reza Jafarzadeh Esfehani, Sahar Sadat Moosavi, Parisa Jalali

**Affiliations:** ^1^Ghaem Hospital, Dr. Shariati Square, Ahmadabad Avenue, Mashhad, Iran; ^2^Student Research Committee, Sabzevar University of Medical Sciences, Sabzevar, Iran; ^3^Azad University of Medical Science, Mashhad, Iran

## Abstract

Idiopathic pulmonary artery aneurysm is a rare condition. This type of aneurysm can be presented with noncardiac symptoms or even asymptomatic. We report a 73-year-old man with a gigantic idiopathic pulmonary artery aneurysm which was referred to our unit for his kidney problems. During his workup we incidentally found the aneurysm by an abnormal chest-X ray and auscultation. Our further evaluations revealed a 9.8 cm aneurysm in transthoracic echocardiography.

## 1. Introduction

Pulmonary artery aneurism (PAA) is a rare condition, mostly arising from main pulmonary artery [1]. The specific prevalence of PAA is unknown, but it was reported in 1 out of every 14000 autopsies [2, 3]. PAA is described as a dilatation of pulmonary artery (PA) [1]. Although there is no accurate definition for PAA, some studies have mentioned 4 cm of diameter as a cut-off point [1]. Subsequent studies reported the upper normal limit of main pulmonary artery (PA) diameter is 29 mm on computed tomography (CT) [4]. PAA usually presents with unspecific symptoms such as dyspnea, hemoptysis, chest pain, and cough [2]. Many medical conditions including congenital heart defects, connective tissue disorders, and pulmonary hypertension can cause PAA [1, 5]. However, idiopathic PAA is an infrequent and rarely reported lesion [3]. Noninvasive imaging techniques including magnetic resonance imaging (MRI) and CT can help clinician in diagnosis, but the gold standard diagnostic tool for PAA is pulmonary angiography [2, 4].

In this case report, we describe a case of huge main PAA with pulmonary valve atresia and right ventricular outlet tract aneurysm (RVOTA).

## 2. Case Presentation

**Figure 1:**
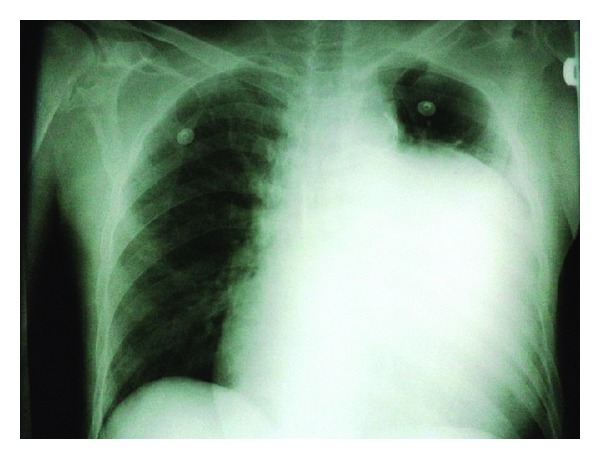
Patient's chest radiography shows cardiothoracic enlargement with homogenous opacity in left hemithorax. Space occupying, cystic lesions, and vascular aneurysm are differential diagnosis of this condition.

**Figure 2:**
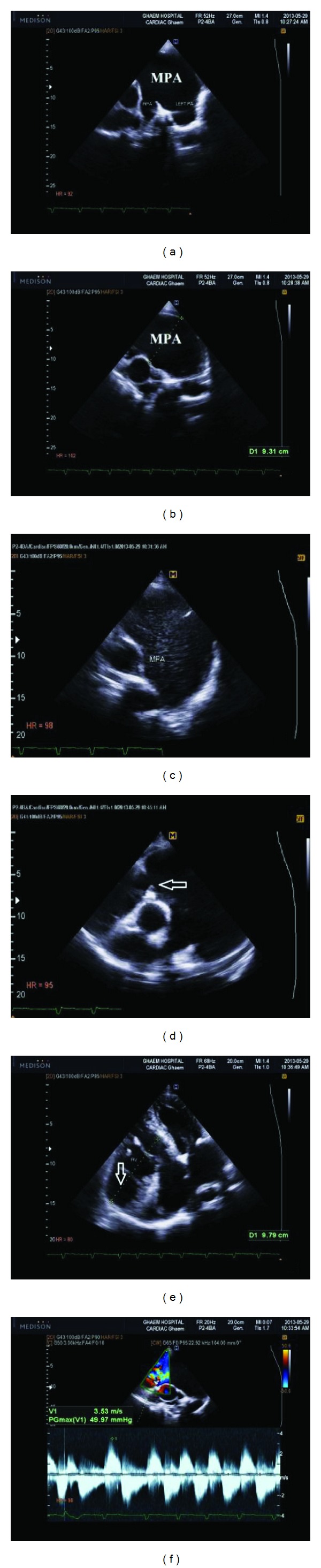
Patient's echocardiography: (a), (b), (c), and (d) parasternal short axis view, visible pulmonary valve (PV), annulus, and retracted leaflets with severe dilatation of main pulmonary artery and both branches (the arrow shows the dysplastic pulmonary leaflet); (e) apical 4 chamber view revealed severe aneurysmal dilatation of right ventricular inflow (the arrow); (f) doppler study of pulmonic valve from high left parasternal view. RPA: right pulmonary artery. PA: pulmonary artery. MPA: main pulmonary artery. RV: right ventricle.

**Figure 3:**
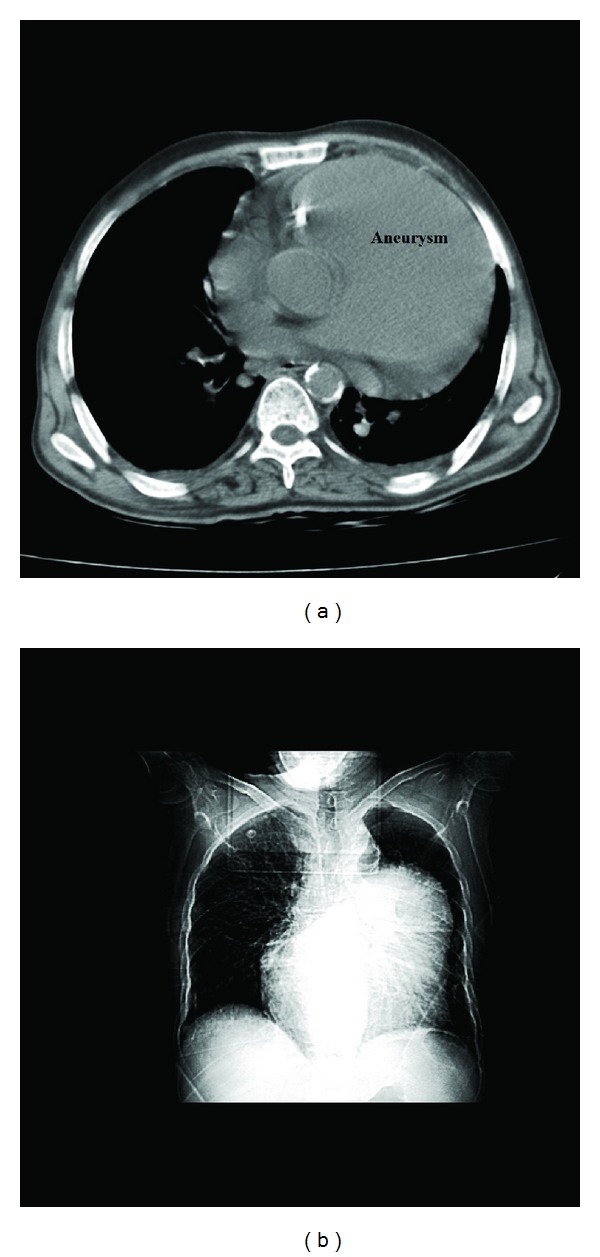
Patient's computed tomography and chest radiography (b) confirmed diagnosis.

On May 2013, a 73-year-old man referred to emergency care unit with shortness of breath, nausea, fever, and chills. His symptoms began from a week ago. In his past medical history he only mentioned a controlled hypertension. His physical examination was normal and only a IV/VI systolic murmur and III/VI diastolic murmur were heard at the left sternal border. His Primary laboratory investigations which was taken by emergency care unit revealed potassium level of 4.7 mEq/dL (normal range between 3.5 and 5.3 mEq/dL), urea level of 66 mg/dL (normal range between 25 and 50 mg/dL), creatinine level of 2 (normal range: up to 1.5 mg/dL), and normal sodium level. His other laboratory tests were all within normal limit. Also, blood and urine cultures were taken in order to exclude infection; a chest X-ray (CXR) and an electrocardiogram (ECG) were also ordered because of his abnormal cardiac auscultation. Because of the high probability of kidney dysfunction and according to his kidney laboratory test results, the patient underwent an abdominal ultrasonography. Ultrasonography revealed a hydronephrotic right kidney and dilated right ureter. On the next day his creatinine level began to rise and his condition worsened. So he underwent urgent dialysis. Till his twentieth day of admission, he underwent dialysis for 6 more times. After 20 days his condition became better and his kidney function tests fell into normal limits. During his kidney work out, because of his abnormal CXR findings (Figure 1) such as cardiothoracic enlargement with a mass lesion in left lung and an abnormal 12 lead EKG with evidences of right ventricular hypertrophy, right axis deviation, and right bundle branch block, a high-resolution computed tomography (HRCT) and an echocardiography were ordered. In the transthoracic echocardiogram (TTE) a pulmonary aneurysm with diameter of 9.8 cm with right ventricular outflow tract (RVOT) dilatation was seen (Figure 2). Also he had severe pulmonary insufficiency (PI) with no pulmonary stenosis (PS) and dysplastic pulmonary leaflet. So, according to his echocardiography result, we decided to take a CT angiography. The CT-angiography results confirmed the diagnosis of PAA and surgical plan was recommended (Figure 3). However, he refused to undergo any surgery and he was discharged with Captopril (CAPTOPRIL, 25 mg Tablet, Irandaru, Tehran) and Carvedilol (CARVIDAL, 12.5 mg Tablet, Alborzdarou, Tehran). He was advised to complete a routine cardiac follow-up each month or whenever he experienced any cardiac or respiratory symptoms such as chest pain or dyspnea.

## 3. Discussion

Idiopathic pulmonary artery aneurysm is mostly diagnosed in autopsy and has high mortality rate [6]. The pathological criteria for idiopathic aneurysm are described as (1) dilation of pulmonary trunk (involvement of arterial tree might or might not be present), (2) absence of extra- or intracardiac shunts, (3) absence of pulmonary disease or chronic cardiac disease, and (4) more than minimal atheromatosis or pulmonary vascular tree arteriosclerosis or absence of arterial disease [7]. According to these criteria the patient had idiopathic pulmonary artery aneurysm. As mentioned before, there is a variation in choosing a normal diameter for pulmonary artery aneurysm. It seems that normal diameters vary upon different patients. These ratios vary between 1.8 cm and 4 cm on CT scan [4, 8]. However, our case (by PAA diameter of 9.8 cm on echocardiography) can be considered as a huge idiopathic aneurysm compared to the highest reported cut-off for PA diameter. Approximately all patients with PAA present with dyspnea and palpitation [1, 3, 7, 9, 10]. In many cases presence of cardiac symptoms such as auscultation abnormalities or abnormal pulmonary artery in chest radiography raised the suspicion of pulmonary artery abnormality and echocardiography, CT scan, and angiography were further performed to confirm the diagnosis [3, 6, 10, 11]. These findings point out the special role of accurate physical examination and inexpensive diagnostic modalities. Various surgical treatments have been reported for PAA in different studies. Kuwaki et al. suggested surgical repair for main pulmonary aneurysms regardless of their underlying disease or etiologies in presence of low operative risk [5]. Several surgical techniques are suggested for the treatment of PAA which include Dacron graft replacement, replacement with combination of Dacron prosthesis and bioprosthesis, aneurysmorrhaphy, and pulmonary allograft repair [5]. As our patient refused to undergo any surgery we decided to arrange regular follow-up visits for him. van Rens et al. study showed that long-term follow-up for several decades is possible [7]. They reported a case of idiopathic left pulmonary aneurysm with a follow-up period of 40 years. They also mentioned that patient monitoring and pulmonary artery checking for regular intervals are probably safe [7].

## 4. Conclusion

There is paucity of literature regarding management of idiopathic PAA. This case report demonstrated the importance of basic physical examination. Idiopathic PAA presents with common cardiac or noncardiac symptoms such as dyspnea or nausea. A simple cardiac examination such as an ECG and careful auscultation can avoid further unnecessary workups and maintain patient's trust in health care providers. The importance of these simple workups in the presence of any other emergency medical conditions should not be neglected.
